# Predicting Soft Tissue Sarcoma Response to Neoadjuvant Chemotherapy Using an MRI-Based Delta-Radiomics Approach

**DOI:** 10.1007/s11307-023-01803-y

**Published:** 2023-01-25

**Authors:** Brandon K. K. Fields, Natalie L. Demirjian, Steven Y. Cen, Bino A. Varghese, Darryl H. Hwang, Xiaomeng Lei, Bhushan Desai, Vinay Duddalwar, George R. Matcuk

**Affiliations:** 1 Department of Radiology & Biomedical Imaging, University of California, San Francisco, San Francisco, CA 94143 USA; 2 College of Medicine – Tucson, University of Arizona, Tucson, AZ 85724 USA; 3 Department of Radiology, Keck School of Medicine of the University of Southern California, Los Angeles, CA 90033 USA; 4 Department of Radiology, S Mark Taper Foundation Imaging Center, Cedars-Sinai Medical Center, 8700 Beverly Blvd., Ste M-335, CA 90048 Los Angeles, USA

**Keywords:** Sarcoma, Soft tissue, Magnetic resonance imaging, Radiomics, Machine learning, Neoadjuvant chemotherapy

## Abstract

**Objectives:**

To evaluate the performance of machine learning–augmented MRI-based radiomics models for predicting response to neoadjuvant chemotherapy (NAC) in soft tissue sarcomas.

**Methods:**

Forty-four subjects were identified retrospectively from patients who received NAC at our institution for pathologically proven soft tissue sarcomas. Only subjects who had both a baseline MRI prior to initiating chemotherapy and a post-treatment scan at least 2 months after initiating chemotherapy and prior to surgical resection were included. 3D ROIs were used to delineate whole-tumor volumes on pre- and post-treatment scans, from which 1708 radiomics features were extracted. Delta-radiomics features were calculated by subtraction of baseline from post-treatment values and used to distinguish treatment response through univariate analyses as well as machine learning–augmented radiomics analyses.

**Results:**

Though only 4.74% of variables overall reached significance at *p* ≤ 0.05 in univariate analyses, Laws Texture Energy (LTE)-derived metrics represented 46.04% of all such features reaching statistical significance. ROC analyses similarly failed to predict NAC response, with AUCs of 0.40 (95% CI 0.22–0.58) and 0.44 (95% CI 0.26–0.62) for RF and AdaBoost, respectively.

**Conclusion:**

Overall, while our result was not able to separate NAC responders from non-responders, our analyses did identify a subset of LTE-derived metrics that show promise for further investigations. Future studies will likely benefit from larger sample size constructions so as to avoid the need for data filtering and feature selection techniques, which have the potential to significantly bias the machine learning procedures.

## Introduction

Assessment of treatment response in soft tissue sarcomas (STS) by conventional radiologic imaging has long posed unique set of challenges for clinicians [1–4]. Owing to their highly variable internal compositions, tumors undergoing a biologic response to chemotherapy may not actually diminish in size due to factors such as cystic degeneration, hyalinization, fibrosis, centralized necrosis, and intratumoral hemorrhage, all of which have the potential to affect estimations of whole-tumor volume [2, 3, 5–10]. Thus, appraisals of treatment response that depend on evaluations of tumor size—including the World Health Organization (WHO) response evaluation criteria and the oft-cited Response Evaluation Criteria In Solid Tumors (RECIST)—may fail to appreciate satisfactory biologic response to chemotherapy in tumors that do not demonstrate macroscopic shrinkage on radiologic imaging [2, 3, 7, 8, 11–16]. The Choi criteria and modified Choi criteria, which were later proposed in an effort to incorporate additional features such as changes in attenuation or signal intensity on CT or MRI, were shown to better correlate with pathologic response [7, 8, 14, 17–19]. However, the Choi criteria were notably not originally designed for STS and still rely heavily on size-based estimations, thus calling into question in their ability to accurately resolve complex architectural changes in STS, particularly in cases of synovial sarcoma [4, 7, 20]. In the age of targeted molecular therapies, there exists a growing need for modernized response criteria that more accurately reflect the scope of phenotypic heterogeneity [6, 13, 21, 22].

Radiomics is defined as the conversion of medical imaging into multi-dimensional mineable data for clinical decision support to bolster accurate diagnosis, prognostication, and prediction of treatment response [4, 23–28]. In comparison with standard biopsy techniques, radiomics analysis offers the advantage of being able to non-invasively quantify heterogeneity of entire tumor volumes at given time points of interest, which in theory should allow for better characterization of chemotherapeutic response than use of size-based criteria alone [6, 20–22, 26, 27, 29–31]. Radiomics has already been successfully applied to a variety of clinical applications related to STS, including stratification of benign from malignant soft tissue neoplasms, prediction of histologic grade, and assessment of metastatic risk [27, 31–34], though lack of standardized protocols has hindered widespread adoption of radiomics workflows in clinical practice [16, 24, 25, 35].

Standard-of-care typically encourages the use of anthracycline-based regimens as first-line chemotherapy in patients with newly diagnosed STS, which have demonstrated improved overall and metastasis-free survival in phase 3 clinical trials [2, 3, 29, 30]. Yet, ongoing research in sarcoma care remains limited in part due to the previously detailed shortcomings of traditional size-based response criteria, which calls into question their appropriateness for use as endpoints in clinical trials [2, 3, 20]. Thus, ongoing collaborations between leading agencies including the US Food and Drug Administration and the US National Cancer Institute have since called for the validation of quantitative imaging techniques to serve as surrogate biomarkers, as these may in fact more accurately reflect early biological changes in tumor physiology [6, 13, 20, 22]. In a previous pilot study [20], we were able to demonstrate that quantitative-MRI (q-MRI) evaluation of enhancing tumor volume was able to accurately stratify responders from non-responders in a small cohort of patients with histopathologically diagnosed STS treated with standard-of-care neoadjuvant chemotherapy (NAC). Therefore, based on studies correlating intratumoral heterogeneity on radiologic imaging with higher histologic grade and poorer patient outcomes [5, 23, 24, 29, 32, 33, 36], we hypothesized that change from baseline of radiomics metrics taken pre- and post-NAC (i.e., delta-radiomics) might be able to better predict response to NAC in STS. While a small body of evidence does suggest a role for radiomics-based predictive modeling in stratifying response to neoadjuvant therapy [30, 34, 37, 38], these studies are not repeatable due to the application of data filtering and feature reduction techniques prior to model training and cross-validation, which has been shown to bias model performance [28, 39]. Thus, we further aimed to investigate whether these previous findings could be replicated under more rigorous test conditions.

## Materials and Methods

This single-center retrospective study was approved by our university’s institutional review board. The requirement for informed consent was waived due to the retrospective nature of our data collection.

### Study Participants

A total of 44 subjects (mean age 53.70 years; range 16–80 years) who received NAC at our institution for histologically diagnosed STS were included in this study. Enrollments were restricted to only subjects who had both a baseline MRI obtained prior to initiation of NAC and a post-treatment MRI obtained at least 2 months following NAC initiation and prior to surgical resection. Patients were identified by chart review of cases discussed at our institution’s Orthopedic and Sarcoma Tumor Boards from January 2010 to January 2017. All 44 subjects were previously reported as part of a related study investigating the utility of radiomics analysis in stratifying benign and malignant soft tissue neoplasms [27]. Seven of these subjects were additionally included in previous pilot studies [1, 20]. In our cohort, the most common pathologic diagnoses were undifferentiated pleomorphic sarcoma (*n* = 17), synovial sarcoma (*n* = 6), myxoid liposarcoma (*n* = 4), and leiomyosarcoma (*n* = 4) (Table 1). Lesions were most often encountered in the thigh (*n* = 21), followed by the arm (*n* = 4), pelvis/buttock (*n* = 4), and calf (*n* = 3).

**Table 1:** Summary features of neoplasms analyzed for machine learning–augmented radiomics analysis

Characteristic	All tumors (*n* = 44)
Age (years)*	53.70 ± 16.05 (16–80)
Sex	
Males	19/44 (43.18%)
Females	25/44 (56.82%)
Anatomic location	
Shoulder	1/44 (2.27%)
Arm	4/44 (9.09%)
Forearm	1/44 (2.27%)
Wrist	1/44 (2.27%)
Groin	2/44 (4.55%)
Hip	1/44 (2.27%)
Pelvis/buttock	4/44 (9.09%)
Thigh	21/44 (47.23%)
Calf	3/44 (6.82%)
Knee	1/44 (2.27%)
Ankle	1/44 (2.27%)
Foot	2/44 (4.55%)
Retroperitoneum	2/44 (4.55%)
Histologic subtypes	
Undifferentiated pleomorphic sarcoma	17/44 (38.64%)
Dedifferentiated liposarcoma	2/44 (4.55%)
Myxoid liposarcoma	4/44 (9.09%)
Pleomorphic liposarcoma	1/44 (2.27%)
Leiomyosarcoma	4/44 (9.09%)
Myxofibrosarcoma	2/44 (4.55%)
Embryonal rhabdomyosarcoma	1/44 (2.27%)
Pleomorphic rhabdomyosarcoma	1/44 (2.27%)
Extraskeletal Ewing sarcoma	2/44 (4.55%)
Extraskeletal osteosarcoma	2/44 (4.55%)
Malignant peripheral nerve sheath tumor	2/44 (4.55%)
Synovial sarcoma	6/44 (13.64%)
Histologic grade	
High	41/44 (93.18%)
Low	3/44 (6.82%)
Surgical margins	
Positive	26/44 (59.09%)
Marginal	7/44 (15.91%)
Negative	6/44 (13.64%)
Not specified	5/44 (11.36%)
NAC response	
Responder	23/44 (52.27%)
Non-responder	21/44 (47.73%)

all data are presented as numerators and denominators with percentages in parentheses unless otherwise specified

^*^Data presented as mean age in years ± standard deviation with range reported in parentheses

*NAC* neoadjuvant chemotherapy

### Sequence Acquisitions

Two MRI studies were analyzed per subject for a total of 88 scans, 37 of which were acquired at our institution and 51 of which were acquired at outside facilities. A total of 29 institutions contributed to the acquisition pool, which featured radiomics data extracted from 11 unique MRI sequences (Table 2). All studies were uploaded to and available for review through our institution’s PACS at the time of subject enrollment.

**Table 2:** Overview of MRI scans obtained at our institution and at outside facilities broken down by plane of section

Sequence	Total (*n* = 88)	Acquired at our institution (*n* = 37)	Acquired at an outside facility (*n* = 51)
T1			
Axial	85.23%	97.30%	76.47%
Coronal	13.64%	2.70%	21.57%
Sagittal	–	–	–
T1 post-contrast			
Axial	5.68%	2.70%	7.84%
Coronal	2.27%	–	3.92%
Sagittal	–	–	–
T1 fat-saturated			
Axial	63.64%	97.30%	39.22%
Coronal	2.27%	–	3.92%
Sagittal	1.14%	–	1.96%
T1 fat-saturated post-contrast			
Axial	84.09%	100.00%	72.55%
Coronal	2.27%	–	3.92%
Sagittal	1.14%	–	1.96%
STIR			
Axial	56.82%	75.68%	43.14%
Coronal	18.18%	8.11%	25.49%
Sagittal	10.23%	10.81%	9.80%
T2			
Axial	13.64%	13.51%	13.73%
Coronal	3.41%	–	5.88%
Sagittal	6.82%	–	11.76%
T2 fat-saturated			
Axial	15.91%	2.70%	25.49%
Coronal	3.41%	–	5.88%
Sagittal	1.14%	2.70%	–
PD			
Axial	6.82%	2.70%	9.80%
Coronal	3.41%	–	5.88%
Sagittal	1.14%	–	1.96%
PD fat-saturated			
Axial	22.73%	8.11%	33.33%
Coronal	–	–	–
Sagittal	4.55%	8.11%	1.96%
HASTE			
Axial	–	–	–
Coronal	1.14%	–	1.96%
Sagittal	–	–	–
FIESTA			
Axial	1.14%	–	1.96%
Coronal	–	–	–
Sagittal	–	–	–

all data are presented as percentages of MRI scans for which the indicated sequence was available for analysis. Unless otherwise specified, all sequences were obtained prior to contrast administration. *STIR* short-tau inversion recovery, *PD* proton density, *HASTE* half-Fourier acquisition single-shot turbo spin-echo, *FIESTA* fast imaging employing steady-state acquisition

### Volumetric Segmentations and Radiomics Data Extraction

**Fig. 1:**
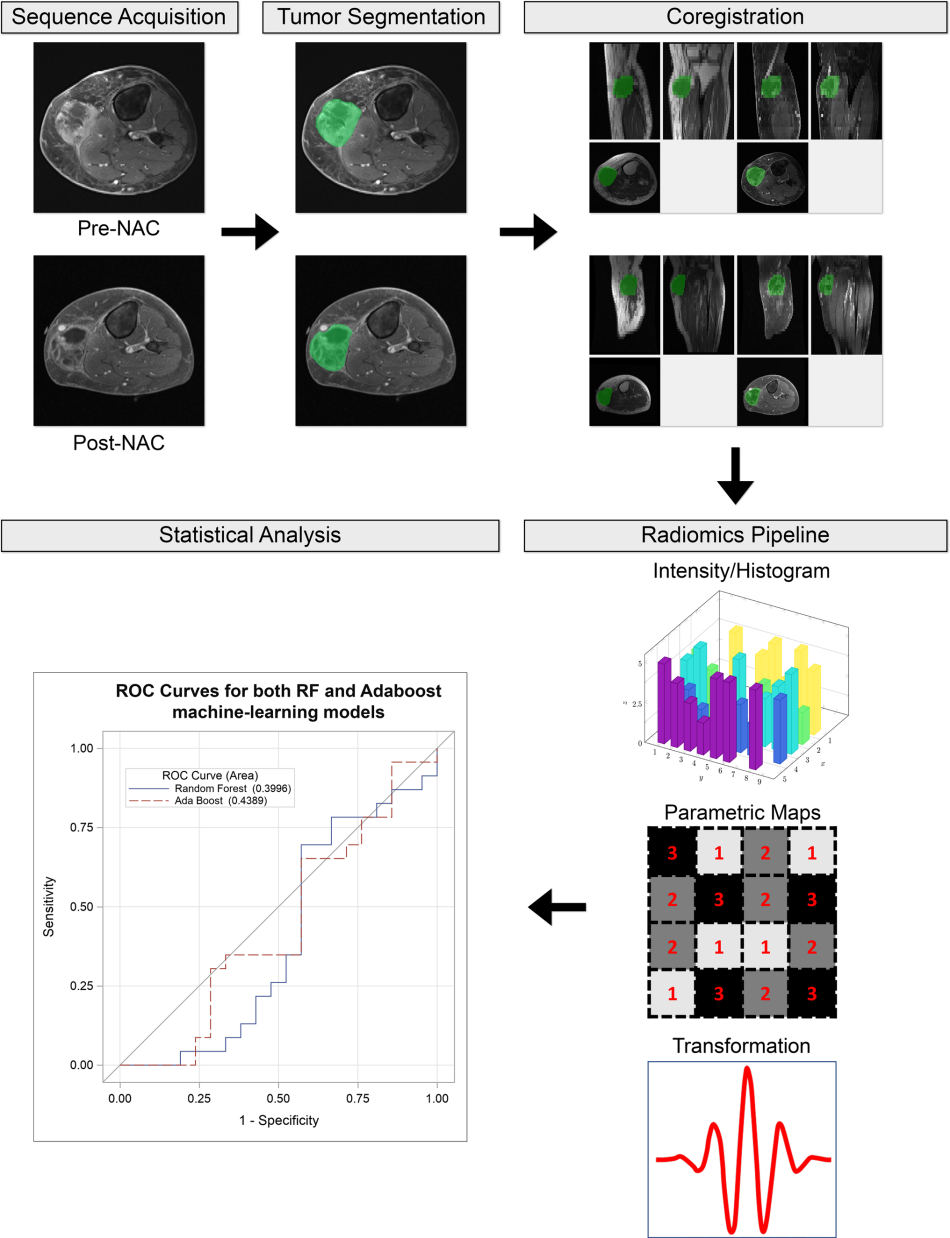
Flowchart illustrating the workflow for radiomics feature extraction and machine learning–augmented analysis. Example depicts a 50-year-old female with undifferentiated pleomorphic sarcoma of the left calf pre- and post-NAC. Following manual segmentation of 3D-ROIs, data were then extracted from co-registered sequences of interest via our institutional radiomics pipeline. Machine learning models using random forest (RF) and real adaptive boosting (AdaBoost) methods were constructed using a tenfold cross-validation procedure. NAC, neoadjuvant chemotherapy; ROI, region of interest; RF, random forest; AdaBoost, real adaptive boosting.

**Fig. 2:**
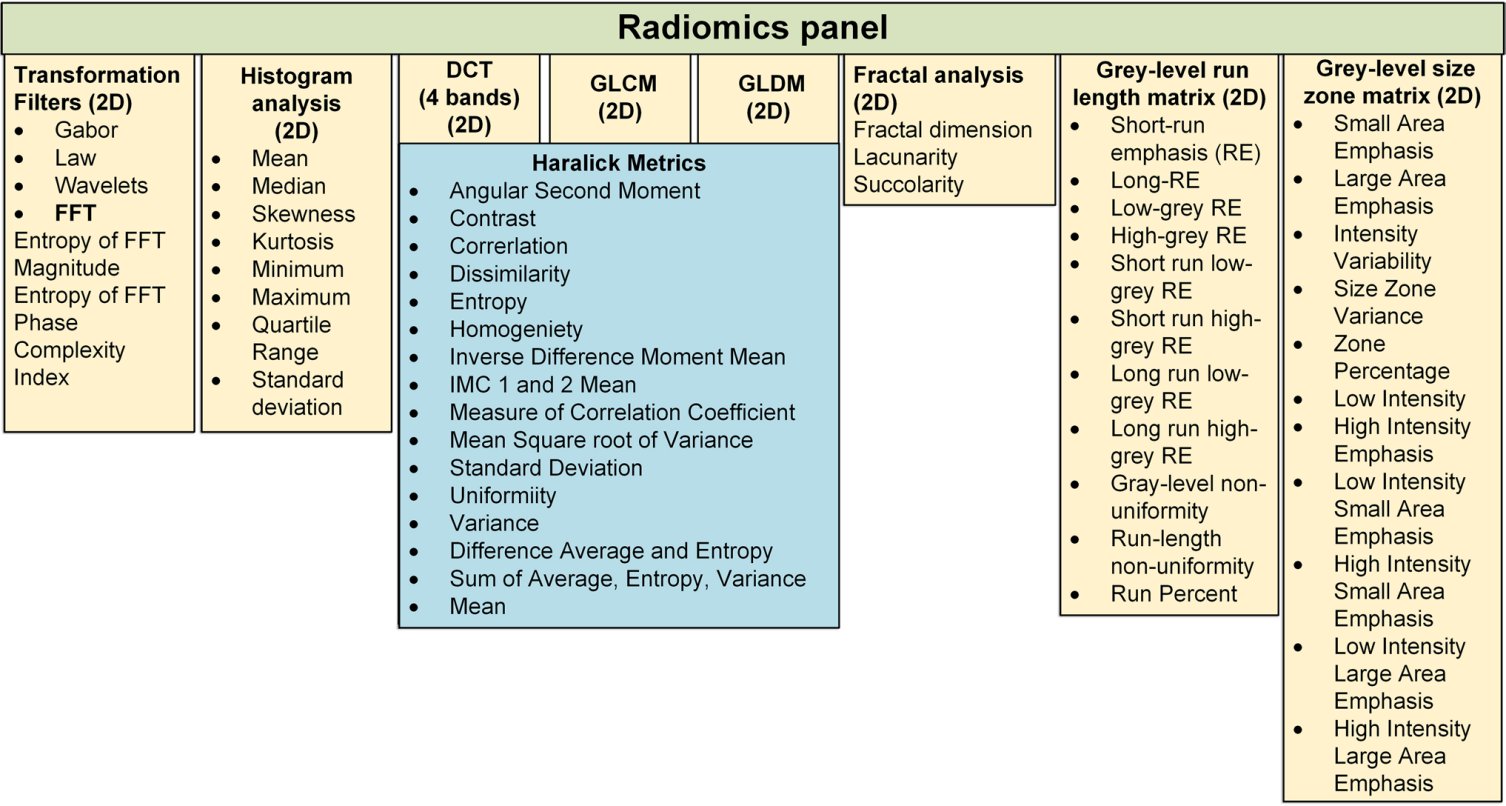
Diagrammatic representation of features extracted by our institutional radiomics pipeline. In total, our radiomics pipeline was engineered to extract 1708 radiomics features from 9 distinct texture families. Reprinted by permission from Springer Nature Customer Service Centre GmbH: Springer Nature European Radiology, [27] (Whole-tumor 3D volumetric MRI-based radiomics approach for distinguishing between benign and malignant soft tissue tumors, Fields et al.), European Society of Radiology (2021).

The workflow for image segmentation and radiomics data extraction has been previously described in detail [27]; briefly, images were loaded onto server-deployed Synapse 3D software (Fujifilm Medical Systems), after which tumor volumes were manually delineated on one sequence of interest. 3D regions of interest (ROIs) were then transferred onto additional sequences of interest from the same MRI study following sequence co-registration using statistical parametric mapping (SPM) software [40] (Fig. 1). Subsequently, 1708 radiomics features were extracted from the 3D-ROIs using MATLAB® (MathWorks) software running our comprehensive institutional radiomics pipeline, which has been rigorously benchmarked against an Image Biomarkers Standardization Initiative (IBSI) phantom and reference values [41] (Fig. 2). Delta values were then calculated from the extracted radiomics features. Generally speaking, delta-radiomics capture either the change or the percent change in radiomics features across different points in time [4, 30, 34, 37, 38]. The quantification of delta change over time in our study was thus calculated as post-NAC minus pre-NAC feature numeric values, from which subsequent analyses and machine learning algorithms were derived.

### Statistical Analyses

Univariate comparisons were performed using independent *t*-test or Wilcoxon rank sum test depending on data normality. Descriptive analyses included mean, standard deviation, and interquartile ranges displayed as box plots. Benjamini–Hochberg Procedure was used to adjust for multiple comparisons errors [42]. The percentages of radiomics features with unadjusted and Benjamini–Hochberg adjusted *p* ≤ 0.05 and *p* ≤ 0.01 within each radiomics family were calculated as an assessment of the overall signal strength of a given family.

Two machine learning algorithms, namely random forest (RF) and real adaptive boosting (AdaBoost), were trained with the aim of distinguishing between responders and non-responders using delta-radiomics values [43]. Both RF and AdaBoost are decision tree-based methods that are robust to non-normal distributions, missing data, and outliers, though RF in particular has performed exceptionally well in related radiomics studies [44–46]. Model performance was evaluated using a tenfold cross-validation. *K*-fold cross-validation is a commonly employed validation technique in radiomics studies [4, 27, 30, 31, 34, 47, 48], as the systematic formulation of multiple training and testing cohorts renders many unanticipated confounders essentially inert. For the purposes of our study, the full dataset was first divided equally into 10 folds. Subsequently, the learning process was re-iterated 10 times, during which a given classifier was applied in turn to each of the testing samples. In this way, each study sample was allowed to serve as an independent test case.

The machine learning models were constructed as previously described [27]. In the case of RF, the model was constructed using 800 trees with a leaf size of 16. Maximal depth was set at 50. The square root of the variable number was taken as the variable to try. Given that AdaBoost is comparatively more efficient, only 25 trees with a maximal depth of 3 were used during model construction [49]. For both models, Gini impurity index served as the loss function. Prior correction as described by King et al. was used to adjust for imbalanced outcomes [50]. The accuracies of the predictive models were quantitatively assessed by taking the areas under the curve (AUCs) of the receiver operating characteristic (ROC) curves generated from the predicted probabilities of the 10 testing datasets combined.

Variables of importance were selected and ranked using out-of-bag Gini index. The cut-off for top performing variables of importance was taken to be the “cliff” of the out-of-bag Gini ranking, i.e., a sudden large change from previous ranking position. The variables of importance selection procedure were repeated 10 times, with the final ranking based on the sum of out-of-bag Gini rankings over the tenfold cross-validation.

SAS Enterprise Miner 15.1: High-Performance Procedures were used for machine learning. SAS v9.4 statistical computing software was used for all other statistical analysis.

## Results

**Fig. 3:**
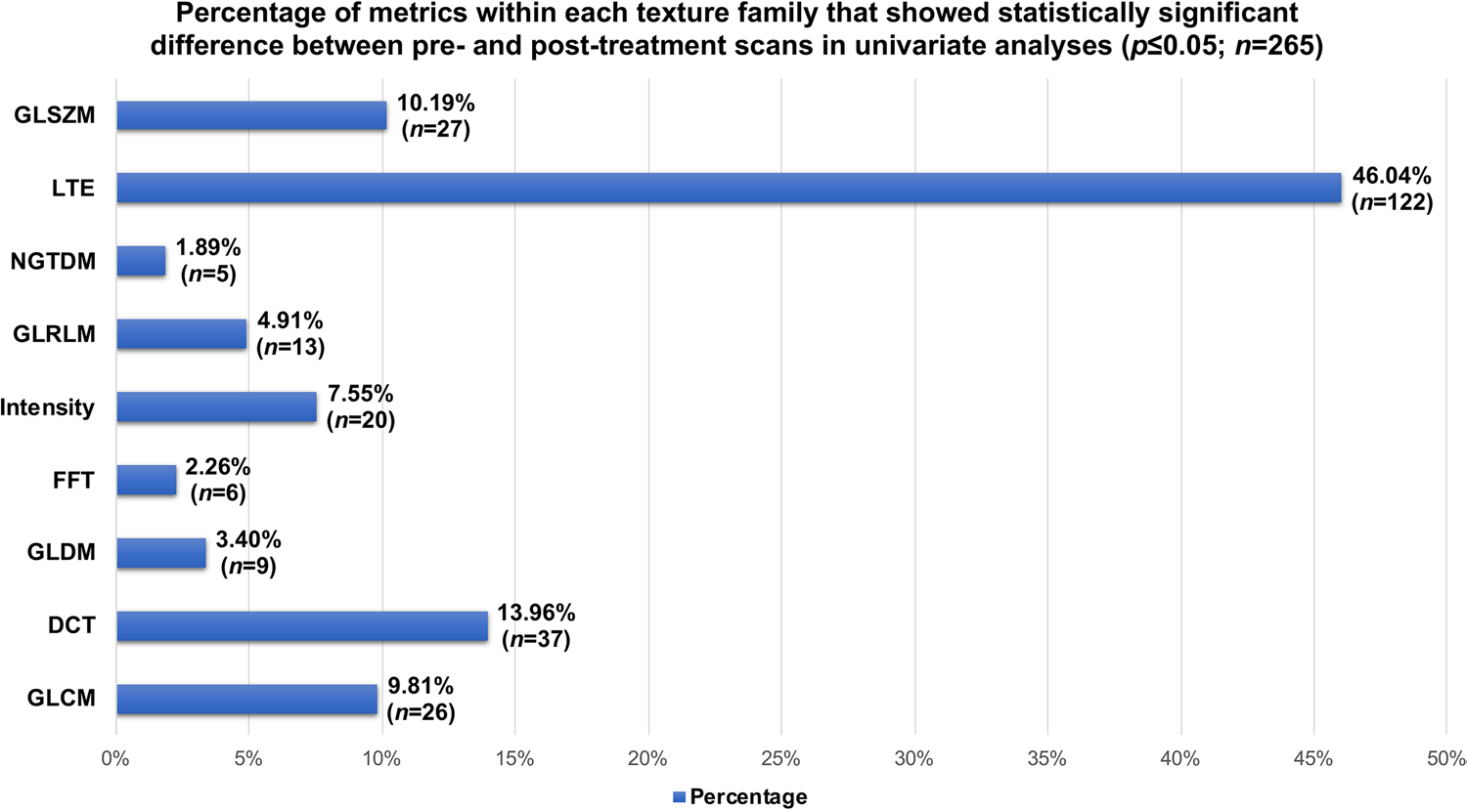
Bar plots illustrating the percentage of radiomics features that were statistically significant at the *p* ≤ 0.05 level broken down by texture family. Overall, only 4.74% of all variables reached statistical significance in univariate analyses; however, analyses did reveal increased representation of LTE-derived features. GLSZM, gray-level size zone matrix; LTE, Laws Texture Energy; NGTDM, neighboring gray tone difference matrix; GLRLM, gray-level run length matrix; FFT, fast Fourier transform; GLDM, gray-level dependence matrix; DCT, discrete cosine transform; GLCM, gray-level co-occurrence matrix.

**Fig. 4:**
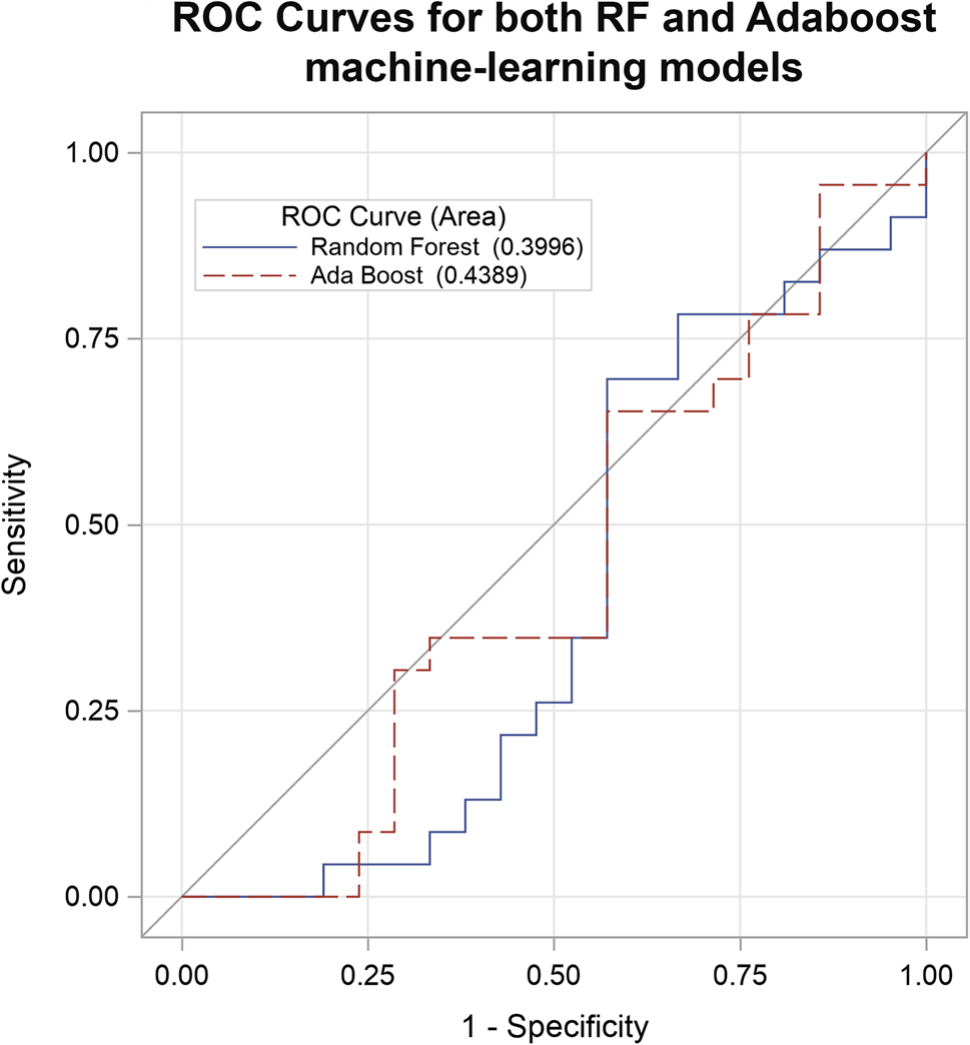
Evaluation of prediction accuracies of the machine learning–augmented decision classifiers using ROC curve analysis. Prediction accuracy was evaluated by AUC. ROC, receiver operating characteristic; AUC, area under the curve; RF, random forest; AdaBoost, real adaptive boosting.

Univariate analyses revealed that only 4.74% (*n* = 265) of variables showed significant differences in delta-radiomics metrics at the *p* ≤ 0.05 level between NAC responders vs. non-responders. Though only a small percentage of metrics overall showed statistical significance, an increased representation of Laws Texture Energy (LTE)-derived features was notably observed as compared to other texture families, accounting for 46.04% (*n* = 122) of all features reaching statistical significance at the *p* ≤ 0.05 level (Fig. 3). Likewise, only 1.34% (*n* = 75) of variables showed statistically significant differences at the *p* ≤ 0.01 level. Concordantly, both machine learning methods failed to predict NAC response by ROC analysis, with AUCs of 0.40 (95% CI 0.22–0.58) and 0.44 (95% CI 0.26–0.62) for RF and AdaBoost, respectively (Fig. 4).

As demonstration of proof of concept, we repeated our machine learning procedure on 2 restricted datasets of pre-selected radiomics features filtered by univariate *p*-values. When run on a restricted dataset of only features found to be significant at the *p* ≤ 0.05 level, RF and AdaBoost yielded AUCs of 0.74 (95% CI 0.59–0.89) and 0.75 (95% CI 0.60–0.89), respectively. Similarly, when run on a restricted dataset of only features found to be significant at the *p* ≤ 0.01 level, RF and AdaBoost yielded AUCs of 0.78 (95% CI 0.64–0.92) and 0.82 (95% CI 0.70–0.95), respectively. This exercise was conducted solely for the purposes of comparison and discussion and is not routinely recommended by the authors as a method of bolstering machine learning results.

## Discussion

MRI remains the preeminent method for serial evaluation of STS progression and treatment response [4, 9, 19, 37, 38]. Yet, it has been clear for some time that historical definitions of treatment response—which have tended to rely on size-based criteria—are severely lacking, as they often fail to account for non-dimensional changes in tumors exhibiting a biologic response to NAC [2–4, 7, 8, 20, 30]. In this study, we investigated the utility of a delta-radiomics approach to predict chemotherapeutic response in STS by assessing for temporal changes in the radiomics feature parameters of MRI scans taken pre- and post-NAC. In contrast to previously published findings [30], we do not find an ability for decision classifiers constructed using an MRI-based delta-radiomics approach to accurately predict treatment response in STS treated with NAC.

Despite a concerted push for the validation of novel therapeutic biomarkers in oncologic care [6, 21, 22], few studies have thus far investigated the utility of MRI-based radiomics features to serve as surrogate predictors of neoadjuvant response in STS [4] (Table 3). To the best of our knowledge, only one previously published study by Crombé et al. similarly utilized an MRI-based delta-radiomics approach for predicting treatment response specifically to NAC. In their procedure, the authors calculated the absolute change in 33 radiomics features in 65 patients with STS following anthracycline-based NAC, from which only a subset of pre-selected delta features was used to train 4 decision classifiers [30]. Likewise, though Peeken et al., Gao et al., and Miao et al. all suggest an ability for delta-radiomics–based decision classifiers to predict STS response to radiotherapy [34, 37, 38], these studies also employed feature reduction or recalling techniques prior to model training. While data filtering has become an unfortunately common practice to address high dimensionality in radiomics datasets, these approaches have the potential to induce information leakage. Information leakage further leads to disruption of test data independency, thereby resulting in problems of overfitting [28, 39]. We demonstrate these phenomena explicitly through the results of our filtered analyses, whereby restricting our machine learning inputs to only variables which were significant at the *p* ≤ 0.05 and *p* ≤ 0.01 levels in our univariate analyses yielded comparable AUCs to those reported by Crombé et al., Peeken et al., Gao et al., and Miao et al. [30, 34, 37, 38].

**Table 3:** Summary of characteristics of comparator studies

First author [reference no.]	Year	Intervention	Sample size	Predictive models investigated	Validation procedure	Highest AUC reported
Crombé [30]	2019	NAC	*n* = 65	RF, support vector machine, k-nearest neighbors, logistic regression	10-fold stratified cross-validation	0.86
Peeken [34]	2021	Neoadjuvant RT with or without NAC	*n* = 156	RF, LogitBoost, elastic net regression	3-fold nested cross-validation	0.75
Gao [37]	2020	Neoadjuvant RT	*n* = 30	Support vector machine, logistic regression	5-fold cross-validation	0.91
Miao [38]	2022	Neoadjuvant RT with or without multi-targeted TKI	*n* = 30	Logistic regression	None	0.92

*NAC* neoadjuvant chemotherapy, *RT* radiotherapy, *AUC* area under the curve, *RF* random forest, *TKI* tyrosine kinase inhibitor

Publication bias has emerged as a growing area of concern among radiomics studies. As recently as 2018, Buvat et al. reported that a mere 6% of all PET radiomics studies in the published literature explicitly reported negative results [51]. Moreover, in a systematic review of 52 sarcoma-specific radiomics studies, Crombé et al. found that no studies specifically described negative findings [36], further highlighting the need for more balanced publication practices within the field. As discussed above, our result was not able to reproduce separation of neoadjuvant responders from non-responders using machine learning augmented MRI-based radiomics analyses [30, 34, 37, 38]. We believe this is in large part due to our more rigorous approach to our machine learning methodologies without reliance on data filtering and feature selection techniques featured in related works [27, 28, 39]. In particular, Crombé et al. even further report that they constructed their models by first selecting one feature per category and then increasing the number of included features in a “forward stepwise fashion” as determined by univariate *p*-values [30]. Such steps are not only unnecessary but actually bias and invalidate the results of modern machine learning approaches such as RF—which was notably their top performing classifier—as these algorithms are designed to work with high dimensionality datasets without pre-selection of so-called candidate features [27, 47, 52].

One other notable aspect of our study’s methodology was our inclusion of scans from multiple image acquisition centers. Issues with reproducibility in radiomics studies has garnered progressively more attention in recent years, as it has become increasingly clear that radiomics-based machine learning procedures based on single-center, single-vendor datasets generalize poorly to multicentric data pools [36, 48, 53, 54]. Moreover, as we have discussed in our prior work [27], databases derived from single-center cohorts are poorly reflective of modern clinical practice models [29, 48]. Thus, our study is in line with literature supporting the use of multicentric datasets in radiomics studies [16, 26, 27, 36, 53, 55], which theoretically would help mitigate confounding effects of signal noise introduced as a result of heterogeneity in acquisition parameters.

Finally, though the results of our machine learning process failed to reach overall statistical significance, we do note an increased representation of LTE-derived metrics in the univariate analyses, with 46.04% of all metrics reaching statistical significance at the *p* ≤ 0.05 level deriving from LTE-based computations. LTE-based measures belong to a group of spatial filtering techniques that reflect the properties of *n x n*-sized “convolution kernels” [56–58]. Using this method, spatial domain filters are generated from the vector products of one-dimensional convolution masks, each representing a different texture feature [58]. In the case of our institutional radiomics pipeline, LTE-based metrics accounted for 1472 individual radiomics features out of a total of 5585 features extracted from 9 separate texture families during the course of this study. This subset of our findings do support previously published data suggesting that spatial filtering techniques are well-suited to detect features indicative of tumor heterogeneity [26, 27], possibly as a consequence of more completely capturing voxel-to-voxel variation through the creation of neighborhood-based matrices [56, 58].

Our study was limited by several factors. First, while our study population was similar in size and composition to the cohort reported on by Crombé et al. [30], it is possible that our study was underpowered to detect a significant result, whereby 100 subjects is often regarded as the threshold sample size for radiomics studies [23]. Although feature selection can theoretically lower the cohort threshold size, we feel that routine use of these procedures should generally be avoided in radiomics studies for reasons as discussed thoroughly above. Thus, given the relative rarity of STS in the general population, multi-institutional collaborations may be necessary in future studies to accrue adequate sample sizes [4, 5, 10, 20, 27, 32, 37, 48]. Second, the retrospective nature of our data collection poses a risk for selection bias given that our subjects were screened for enrollment eligibility from a larger pool of cases discussed at our institution’s Orthopedic and Sarcoma Tumor Boards [59]. Third, though efforts are currently being made to standardize post-acquisition harmonization techniques [10, 24, 31, 60, 61], such applications lack general consensus regarding proper implementation and execution [26, 27]. Furthermore, while post-processing data harmonization techniques such as ComBat have shown some ability to ameliorate scanner and protocol variabilities in multicentric studies, such batch adjustment methods have limitations when used in small sample sizes and rely on stringent data distribution assumptions. [62]. Thus, these methods were of limited applicability to our dataset given concerns for adverse effects due to outliers as well as missing and skewed data distributions. Future efforts to validate post-processing methods aimed at mitigating signal instability across heterogeneous acquisition parameters will undoubtedly aid in the construction of large, multicentric datasets for future research. Additional future directions may also include focused studies correlating delta-radiomics changes with histologic subtype and histopathologic findings of percent necrosis, as well as those specifically focused on stratifying post-treatment changes related to specific chemotherapeutic regimens.

In conclusion, though our machine learning analyses did not show statistically significant separation of NAC responders from non-responders, we were able to identify increased representation of LTE-derived metrics in univariate analyses. These and other spatial filtering metrics may pose a promising area for future radiomics research due to their ability to more accurately reflect subtle variations in the imaging grayscale [26, 27, 56, 58]. Larger sample sizes in future cohorts are warranted so as to obviate the need for data reductive techniques, which carry with them an inherent risk of introducing information leakage and thus biasing the decision classifiers [28, 39].

## Abbreviations

STS: Soft tissue sarcoma
RECIST: Response Evaluation Criteria In Solid Tumors
NAC: Neoadjuvant chemotherapy
RF: Random forest
AdaBoost: Real adaptive boosting
AUC: Area under the curve
ROC: Receiver operating characteristic
LTE: Laws Texture Energy
